# Association between noise pollution perception and prevalence of cardiovascular disease in a Chinese adult population: a cross-sectional study based on the CGSS 2021 cardiovascular disease dataset

**DOI:** 10.3389/fpubh.2026.1873102

**Published:** 2026-06-29

**Authors:** Ziyun Zhang, Peng Shi, Zili Zuo

**Affiliations:** 1 School of Life and Science, Huzhou College, Huzhou, China; 2 School of Physical Education, Shandong University of Technology, Zibo, China

**Keywords:** cardiovascular disease, China, health, noise pollution, subjective perception

## Abstract

**Objective:**

To explore the association between Chinese adults' subjective perception of noise pollution and the prevalence of cardiovascular diseases, so as to make up for the deficiencies in data and perspectives of previous studies and provide a basis for formulating relevant prevention and control strategies.

**Methods:**

A total of 2,717 relevant records from the 2021 China General Social Survey (CGSS 2021) cardiovascular disease dataset were used. The core independent variable was the degree of noise pollution perceived by residents themselves (low pollution, high pollution), and the dependent variable was whether they suffered from cardiovascular diseases (including hypertension, dyslipidemia, heart disease, and stroke). Data processing and statistical analysis were conducted through chi-square test and binary Logistic regression analysis using software such as SPSS 25.0 and Stata 12.0.

**Results:**

There was no statistically significant difference in the prevalence of overall cardiovascular diseases (χ^2^ = 0.083, *P* = 0.773), hypertension (χ^2^ = 0.790, *P* = 0.374), dyslipidemia (χ^2^ = 2.063, *P* = 0.151), heart disease (χ^2^ = 2.596, *P* = 0.107) and stroke (χ^2^ = 3.748, *P* = 0.053) between the group with high noise pollution perception and the group with low noise pollution perception. However, after controlling for confounding factors, the group with higher noise pollution perception had a higher prevalence of heart disease compared with the group with lower noise pollution perception (*OR* = 1.751, 95% *CI* = 1.203~2.550, *P* < 0.01).

**Conclusion:**

There is a significant positive association between Chinese adults' subjective perception of noise pollution and the prevalence of heart disease. This suggests that when formulating strategies for noise pollution prevention and control and cardiovascular disease prevention, both objective data and residents' subjective perceptions need to be considered.

## 1 Introduction

With the acceleration of urbanization, noise pollution has become an increasingly serious environmental problem (1). Noise pollution not only interferes with people's daily lives, work, and study, but also triggers a range of physiological and psychological problems, seriously threatening public health and quality of life. Relevant studies (2–5) has pointed out that long-term exposure to noisy environments can have multiple negative impacts on human health, including the cardiovascular system, the nervous system, and the auditory system. A report (6) released by the European Environment Agency indicates that noise pollution has become an important environmental health issue in Europe, with road noise being the main source. More than 125 million Europeans are troubled by road noise above 55 decibels. On the contrary, a study (7) has pointed out that reducing noise by 5 decibels can lower the prevalence of high blood pressure in the United States by 1.4%, the prevalence of coronary heart disease by 1.8%, and increase annual economic benefits by 3.9 billion US dollars. Therefore, noise pollution not only has negative impacts on the physical and mental health of the nation, but also creates an economic burden for the development of the country and society.

Meanwhile, cardiovascular disease has become one of the leading causes of death and disability worldwide (8). Hu et al. (9) analyzed the temporal trends in the global burden of cardiovascular diseases from 1990 to 2021 and projected the future trends up to 2045. They found that the absolute numbers of deaths and disability-adjusted life years (DALYs) due to cardiovascular diseases increased significantly from 1990 to 2021. In addition, the prediction model revealed that the absolute numbers of deaths and DALYs will continue to rise from 2022 to 2045. In addition, compared with 1990, the incidence, prevalence and mortality of cardiovascular diseases in China showed an increasing trend year by year in 2019, with the incidence, prevalence and mortality increasing by 93.75%, 99.75% and 57.39% respectively (10). The high incidence, high mortality and high disability rate of cardiovascular diseases have brought a heavy burden to society and families.

A growing body of studies (11–13) has shown that there is a close association between noise pollution and cardiovascular diseases. For example, Osborne et al. (13) found that traffic noise exposure was significantly positively associated with the incidence of adverse cardiovascular events. In addition, a systematic review and meta-analysis by Rabiei et al. (14) revealed a significant association between noise exposure and cardiovascular diseases. Long-term exposure to a noisy environment can enhance the body's stress response, stimulate the excitation of the sympathetic nervous system, and trigger the release of stress hormones such as norepinephrine (11). This, in turn, leads to physiological changes like increased blood pressure, accelerated heart rate, and vasoconstriction (13). These changes will increase the burden on the heart, affect the normal function of the cardiovascular system, and may cause the occurrence and development of cardiovascular diseases after long-term accumulation (11, 13).

Currently, research on the relationship between noise pollution and cardiovascular diseases still has some limitations. On the one hand, most studies focus on the relationship between occupational or specific environmental noise and cardiovascular health, whereas research on the association between common daily environmental noise, particularly subjectively perceived noise pollution and cardiovascular disease remains limited. On the other hand, previous studies mostly used objectively measured noise data. However, individuals' subjective perception of noise is the key factor triggering physiological and psychological responses. Factors such as an individual's sensitivity to noise, adaptability, and psychological state can lead to different subjective feelings about the same noise level, which in turn have varying degrees of impact on the cardiovascular system. Therefore, relying solely on objective noise measurement data makes it difficult to comprehensively and accurately assess the association between noise pollution and cardiovascular health.

This study aims to explore the association between residents' self-perceived noise pollution and the prevalence of cardiovascular diseases among Chinese adults based on the 2021 China General Social Survey (CGSS) cardiovascular disease dataset. The innovation of this study lies in the first use of the CGSS 2021 cardiovascular disease dataset to investigate the association between noise pollution and cardiovascular diseases from the perspective of subjective perception, which makes up for the deficiencies in data and research perspectives in previous studies. Meanwhile, various advanced statistical analysis methods are adopted to comprehensively control potential confounding factors and conduct an in-depth analysis of the association between the two. This is expected to provide more scientific and comprehensive evidence for research on noise pollution and cardiovascular diseases, and offer new theoretical basis and practical guidance for formulating effective noise pollution prevention and control strategies as well as cardiovascular disease prevention measures.

## 2 Methods

### 2.1 Study design

This study is a cross-sectional observational study based on data analysis from the public CGSS 2021 database. The cross-sectional design allows for a rapid assessment of the association between noise pollution perception and the prevalence of cardiovascular diseases at a specific time point, providing preliminary epidemiological evidence for the core research question of this study.

### 2.2 Participants

As the earliest national, comprehensive, and continuous academic survey project launched in China, the CGSS has established a unique data collection system. Its database, characterized by systematicness and comprehensiveness, collects data across multiple levels such as society, community, family, and individual, providing solid support for analyzing the inherent laws of social changes and exploring major issues with both scientific value and practical significance (15). In terms of sample selection, CGSS adopts a rigorous multi-stage stratified random sampling strategy, covering 31 provincial administrative units in mainland China. In specific operations, contact is first established with respondents via telephone, followed by on-site visits to complete information collection (16). The sampling structure is divided into three levels: residential areas or counties serve as the first-stage sampling units, villages or urban neighborhood communities as the second-stage sampling units, and families as the third-stage sampling units, with only one person selected from each family to participate in the survey (17). The entire sampling process is stratified according to socio-economic and demographic indicators, and the sampling probability is in direct proportion to the size (17). It is worth noting that CGSS 2021 conducted the first comprehensive and detailed special survey on Chinese residents' perception of noise pollution and their prevalence of cardiovascular diseases, providing highly valuable empirical data for the development of this research. The database of that year contains 2717 relevant records, which have become the core analysis objects of this study. In the data preprocessing stage, the research team eliminated invalid responses such as “don't know” and “not applicable” as well as missing data, and used the linear interpolation method to supplement the missing values. Nowadays, CGSS data has become a core data source for studying Chinese society and is widely used in fields such as scientific research, teaching, and government decision-making. Existing studies have explored environmental pollution issues in China using CGSS data (18), fully confirming the comprehensiveness and reliability of its data.

### 2.3 Variables and tools

#### 2.3.1 Independent variable

In this study, noise pollution is set as the core independent variable. CGSS 2021 conducted a survey on the degree of noise pollution in the residential areas of the respondents using a four-level self-assessment scale. The original scoring criteria of the scale are as follows: 1 represents “very serious” noise pollution, 2 represents “relatively serious”, 3 represents “not too serious”, and 4 represents “not serious at all”. To facilitate subsequent statistical analysis, the research team re-coded the original scores. The specific adjustment rules are: the original code 1 is corresponding to 4, with the corresponding description still being “very serious”; the original code 2 is corresponding to 3, with the description adjusted to “serious”; the original code 3 is corresponding to 2, with the description changed to “not serious”; the original code 4 is corresponding to 1, with the description changed to “not serious at all”. After the re-coding, the study further merged the coded values: codes 1 and 2 are classified into the “low pollution” category, and codes 3 and 4 are classified into the “high pollution” category. It is worth noting that existing studies have confirmed that there is a high association between the environmental pollution degree perceived by respondents and the environmental pollution data directly measured by objective means (19). This measurement method based on subjective perception is also widely used in relevant studies in the field of environmental health promotion (20, 21).

#### 2.3.2 Dependent variables

This study identifies the prevalence of cardiovascular diseases as the dependent variable. In the CGSS 2021 survey, respondents were asked separately whether they suffered from hypertension, dyslipidemia, heart disease, and stroke, with “1” representing “yes” and “2” representing “no” in the questionnaire. All disease items require respondents to report physician-diagnosed conditions, rather than making subjective judgments by themselves. To facilitate subsequent statistical analysis, the research team recoded these original data: the original code “1” remains unchanged, still representing “yes”; the original code “2” is adjusted to “0” to indicate “no”. On this basis, the study constructs the variable of “cardiovascular disease status”. Specifically, according to the respondents' answers, if a person suffers from any one of hypertension, dyslipidemia, heart disease, or stroke, this group is assigned a value of “1”, meaning they have cardiovascular diseases; for the group that does not suffer from any of the above diseases, they are assigned a value of “0”, indicating they do not have cardiovascular diseases. Practice has proved that this self-reporting method has high reliability and validity. It can not only effectively promote communication between doctors and patients, but also help optimize symptom management and provide a reliable basis for clinical decision- making (22).

#### 2.3.3 Control variables

Drawing on relevant previous research findings (23, 24), this study selects age, gender, location, residence, education level, body mass index (BMI), and socio-economic status (SES) as control variables. (1) Age: CGSS 2021 surveyed the date of birth of the subjects, and this study obtained their age data by calculating the difference between 2021 and the participants' year of birth. (2) Gender: In this study, males are marked as 1 and females as 2. (3) Location: CGSS 2021 obtained information about the provinces where the subjects are located. This study divides China's provinces into eastern, central and western regions in accordance with the standards for dividing China's geographical regions (25), and assigns them the values 1, 2, and 3 respectively. Among them, the eastern region includes 12 provinces, municipalities and autonomous regions: Beijing, Tianjin, Hebei, Liaoning, Shanghai, Jiangsu, Zhejiang, Fujian, Shandong, Guangdong, Guangxi, and Hainan; the central region includes 9 provinces and autonomous regions: Shanxi, Inner Mongolia, Jilin, Heilongjiang, Anhui, Jiangxi, Henan, Hubei, and Hunan; the western region includes 10 provinces, municipalities and autonomous regions: Chongqing, Sichuan, Guizhou, Yunnan, Tibet, Shaanxi, Gansu, Qinghai, Ningxia, and Xinjiang. (4) Residence: In this study, living in urban areas is assigned a value of 1, and living in rural areas is assigned a value of 2. (5) Education level: Referring to previous studies (26), this study classifies “no education, private school, literacy class, primary school” into one category, assigned a value of 1, that is, “primary school and below”; “junior school” is a separate category, assigned a value of 2; “vocational high school, general high school, technical secondary school, technical school” are merged into one category, assigned a value of 3, that is, “senior school”; “junior college, undergraduate, postgraduate and above” are combined into one category, assigned a value of 4, that is, “college and above”. (6) BMI: CGSS 2021 collected information on the height and weight of the respondents, and this study calculated the BMI values of the subjects according to the formula BMI = weight (kg) / height (m)^2^. (7) SES: CGSS 2021 uses a ten-level scoring method to investigate the current social class of the subjects. The higher the score, the higher the SES.

### 2.4 Mathematical statistics

In this study, SPSS 25.0 and Stata 12.0 software were used for data processing and statistical analysis, and GraphPad Prism 8 software was employed for visualizing the statistical results. Descriptive statistics for continuous variables were presented as mean (*M*) and standard deviation (*SD*); for categorical variables, they were expressed as frequency and percentage (%). Firstly, the contingency table χ^2^ test was used to explore the association between noise pollution and the prevalence of cardiovascular diseases. Secondly, binary Logistics regression analysis was applied to investigate the association between noise pollution and the prevalence of cardiovascular diseases after controlling for confounding factors. Before the regression analysis, multicollinearity was examined using the variance inflation factor (VIF). The results showed that the VIF values of all variables were less than 5, indicating no significant multicollinearity and satisfying the prerequisite assumptions for regression analysis. This study mainly focused on the independent association between noise pollution perception and cardiovascular morbidity; therefore, no interaction effect tests were performed. In addition, linear interpolation was used to impute missing values in the dataset. The significance level for all statistical methods involved in this study was set at α = 0.05.

## 3 Results

### 3.1 Demographic information of participants

Among the 2,717 participants involved in this study, there is a certain span in the age distribution. The average age is 52.04 years, with a standard deviation of 17.64 years, which indicates that the age range of the sample is relatively wide. In terms of regional origin, the distribution of participants shows a pattern of more in the east and fewer in the west. Among them, the proportion of those from the eastern region is the highest, reaching 45.4%; the central region comes next, accounting for 34.6%; and the participants from the western region are relatively fewer, with a proportion of 20.0%. In terms of urban-rural composition, urban residents are slightly in the majority, accounting for 55.9%, while rural residents account for 44.1%. In terms of gender ratio, the proportion of female participants is higher, at 54.8%, and males account for 45.2%. The distribution of educational attainment shows that the proportion of people in the basic education stage is relatively large. Participants with primary school education or below account for 33.4%, and those with junior high school education account for 28.3%. In terms of perception of noise pollution, most participants believe that the noise pollution level in their living environment is low, and this group accounts for as high as 77.6%; only 22.4% of participants perceive high noise pollution. It is worth noting that among these participants, the prevalence of cardiovascular diseases is about 20.6%. Specifically, for various cardiovascular diseases, the prevalence of hypertension is the highest, reaching 17.2%; heart disease follows closely, accounting for 6.5%; the prevalence of dyslipidemia is 3.6%; and the prevalence of stroke is the lowest, only 0.8%. For more detailed demographic information about the participants, please refer to the specific content in Table 1.

**Table 1:** Demographic information of participants.

Continuous variables	*M*	*SD*	Categorical variable	Frequency	%
Age	52.04	17.64	**Gender**		
BMI (kg/m^2^)	23.07	3.74	Male	1,228	45.2
SES	4.28	1.81	Female	1,489	54.8
**Categorical variable**	**Frequency**	**%**	**Location**		
**Education level**			Eastern	1,234	45.4
Primary school and below	907	33.4	Central	940	34.6
Junior school	770	28.3	Western	543	20.0
Senior school	489	18.0	**Residence**		
College and above	551	20.3	Urban	1,518	55.9
			Rural	1,199	44.1

*M*, mean; *SD*, standard deviation; BMI, body mass index; SES, socio-economic status. Black font indicates categorical variables.

### 3.2 Analysis of intergroup comparisons of the prevalence of cardiovascular diseases

An analysis from a statistical perspective on the differences in health indicators between groups with different perceptions of noise pollution reveals the following: When comparing the two groups of people who perceive their environment as having low pollution vs. high pollution, both the overall prevalence of cardiovascular diseases (χ^2^ = 0.083, *P* = 0.773) and the prevalence rates of specific conditions such as hypertension (χ^2^ = 0.790, *P* = 0.374), heart disease (χ^2^ = 2.596, *P* = 0.107), dyslipidemia (χ^2^ = 2.063, P = 0.151), and stroke (χ^2^ = 3.748, *P* = 0.053) show that the *P*-values from all the tests are greater than 0.05. This indicates that the differences in the prevalence rates of the aforementioned diseases between the two groups do not reach a statistically significant level. For a detailed comparison of the data, please refer to Table 2.

**Table 2:** Analysis of intergroup comparisons of the prevalence of cardiovascular diseases.

Variables	Low pollution (*n*/%)	High pollution (*n*/%)	χ^2^	*P*
Cardiovascular diseases				
Affected	438 (20.77)	123 (20.23)	0.083	0.773
Not affected	1,671 (79.23)	485 (79.77)		
Hypertension				
Affected	369 (17.50)	97 (15.95)	0.790	0.374
Not affected	1,740 (82.50)	511 (84.05)		
Heart disease				
Affected	128 (6.07)	48 (7.89)	2.596	0.107
Not affected	1,981 (93.93)	560 (92.11)		
Dyslipidemia				
Affected	71 (3.37)	28 (4.61)	2.063	0.151
Not affected	2,038 (96.63)	580 (95.39)		
Stroke				
Affected	14 (0.66)	9 (1.48)	3.748	0.053
Not affected	2,095 (99.34)	599 (98.52)		

### 3.3 The relationship between noise pollution and the prevalence of cardiovascular diseases

After controlling for relevant variables, compared with the group with low pollution perception, the group with high pollution perception had a higher prevalence of heart disease (*OR* = 1.751, 95% *CI* = 1.203~2.550, *P* < 0.01). However, there was no significant (*P* > 0.05) association between the two groups in terms of the prevalence of cardiovascular diseases (*OR* = 1.264, 95% *CI* = 0.974~1.641), hypertension (*OR* = 1.126, 95% *CI* = 0.850~1.492), dyslipidemia (*OR* = 1.573, 95% *CI* = 0.978~2.529), and stroke (*OR* = 2.260, 95% *CI* = 0.930~5.503). Regarding the relevant control variables, age showed a significant positive association with the prevalence of cardiovascular diseases, hypertension, heart disease, dyslipidemia, and stroke (*P* < 0.01). Compared with urban residents, rural residents had a lower prevalence of stroke (*P* < 0.05). Compared with residents with education level of primary school or below, residents with junior high school education had a lower prevalence of stroke (*P* < 0.05), and residents with college education or above had lower prevalence of cardiovascular diseases and hypertension (*P* < 0.05). BMI was significantly positively correlated with the prevalence of cardiovascular diseases, hypertension, heart disease, and dyslipidemia (*P* < 0.01). SES was significantly negatively correlated with the prevalence of cardiovascular diseases, heart disease, and dyslipidemia (*P* < 0.05). There was no significant association between gender or place of residence and the prevalence of cardiovascular diseases (*P* > 0.05). The results of the binary logistic regression analysis on the relationship between noise pollution and the prevalence of cardiovascular diseases are shown in Table 3.

**Table 3:** Results of binary logistic regression analysis on the relationship between noise pollution and the prevalence of cardiovascular diseases.

Variables	Cardiovascular diseases		Hypertension		Heart disease		Dyslipidemia		Stroke	
	*OR*	95% *CI*	*OR*	95% *CI*	*OR*	95% *CI*	*OR*	95% *CI*	*OR*	95% *CI*
Noise pollution										
High pollution	1.264	(0.974, 1.641)	1.126	(0.850, 1.492)	1.751^**^	(1.203, 2.550)	1.573	(0.978, 2.529)	2.260	(0.930, 5.503)
**Age**	1.081^**^	(1.071, 1.091)	1.082^**^	(1.072, 1.093)	1.076^**^	(1.062, 1.091)	1.062^**^	(1.044, 1.079)	1.051^**^	(1.016, 1.086)
Gender										
Male	1.090	(0.878, 1.352)	1.043	(0.828, 1.313)	1.349	(0.965, 1.886)	1.211	(0.786, 1.865)	0.746	(0.318, 1.749)
Location										
Central	1.012	(0.793, 1.292)	0.955	(0.737, 1.239)	0.900	(0.617, 1.314)	0.968	(0.595, 1.576)	2.161	(0.791, 5.906)
Western	1.083	(0.809, 1.450)	0.972	(0.710, 1.330)	1.129	(0.731, 1.744)	1.196	(0.675, 2.117)	2.145	(0.677, 0.674)
Residence										
Rural	0.913	(0.720, 1.157)	0.868	(0.674, 1.117)	1.137	(0.790, 1.636)	0.858	(0.530, 1.387)	0.311^*^	(0.116, 0.834)
Education level										
Junior school	0.925	(0.713, 1.199)	0.844	(0.639, 1.113)	1.077	(0.727, 1.595)	0.719	(0.412, 1.257)	0.273^*^	(0.076, 0.983)
Senior school	0.879	(0.629, 1.227)	0.844	(0.592, 1.203)	0.870	(0.506, 1.498)	1.201	(0.642, 2.247)	0.311	(0.065, 1.485)
College and above	0.609^*^	(0.383, 0.969)	0.494^**^	(0.294, 0.831)	0.806	(0.378, 1.717)	1.372	(0.612, 3.076)	0.494	(0.089, 2.739)
**BMI**	1.120^**^	(1.088, 1.154)	1.135^**^	(1.100, 1.171)	1.102^**^	(1.054, 1.152)	1.201^**^	(1.136, 1.271)	1.015	(0.911, 1.301)
**SES**	0.925^**^	(0.874, 0.978)	0.944	(0.889, 1.002)	0.917^*^	(0.840, 0.999)	0.871^*^	(0.777, 0.977)	0.814	(0.644, 1.029)

BMI, body mass index; SES, socio-economic status. ^*^P < 0.05; ^**^ *P* < 0.01.

## 4 Discussions

Based on the cardiovascular disease dataset from CGSS 2021, this study explored the relationship between noise pollution perception and the prevalence of cardiovascular diseases among Chinese adults. It was found that the group with a higher perception of noise pollution had a higher prevalence of heart disease. In addition, no significant association was observed between noise pollution perception and the prevalence of hypertension, dyslipidemia, stroke, or overall cardiovascular diseases.

### 4.1 Positive association between noise pollution perception and prevalence of heart disease

The results of this study found that the group with a high perception of noise pollution has a higher prevalence of heart disease, which is consistent with previous studies (27–29). All these studies suggest that higher levels of noise pollution are associated with the occurrence and progression of heart disease. There are multiple potential reasons behind this phenomenon, which can be comprehensively explained from physiological, psychological, and behavioral perspectives.

As a physical environmental stimulus, noise can directly activate the body's stress system, triggering a series of physiological chain reactions such as sympathetic nerve excitation and hormonal disorders (30, 31), oxidative stress and inflammatory responses (32, 33), and decreased sleep quality (34). Prolonged exposure to a high-noise environment keeps the human sympathetic nervous system in a state of persistent excitement, leading to increased secretion of catecholamine hormones such as adrenaline and noradrenaline, which in turn causes accelerated heart rate, elevated blood pressure, and vasoconstriction (30, 31). Such persistent blood pressure fluctuations and abnormal vascular tension can damage vascular endothelial cells and increase the risk of heart disease (35). Noise stimulation can induce the body to produce excessive reactive oxygen species, triggering oxidative stress responses, while activating the release of inflammatory factors (such as interleukin-6, tumor necrosis factor-α, etc.) (32, 33, 36, 37). Oxidative stress and chronic inflammatory states can further exacerbate vascular damage, disrupt the homeostasis of the cardiovascular system, and lay hidden risks for the occurrence of heart disease (38). In addition, noise is a significant factor that disrupts sleep. Long-term sleep interruption or poor sleep quality can prevent the body from carrying out normal repair and regulation processes (34). Studies (39, 40) have shown that insufficient sleep may directly impair the function of myocardial cells and increase the risk of heart disease.

Pollution perception reflects individuals' subjective judgment of environmental risks, and such subjective cognition may be indirectly associated with the prevalence of heart disease through psychological states and behavioral patterns. Individuals who are more sensitive to noise pollution are more likely to experience negative emotions such as health concerns and anxiety (41). Long-term negative emotions may further aggravate hormonal imbalance via the hypothalamic-pituitary-adrenal (HPA) axis, which is associated with reduced capacity for cardiovascular repair (42, 43). In addition, some individuals with higher pollution perception may reduce outdoor activities due to environmental worries, resulting in insufficient physical activity (61). Physical inactivity is a well-recognized risk factor for heart disease; it is associated with declined cardiopulmonary function and elevated risks of obesity and metabolic syndrome, which in turn are related to a higher prevalence of heart disease (44, 45).

To sum up, noise pollution directly damages the cardiovascular system through physiological stress, while “high pollution perception” amplifies such damage through psychological states and behavioral patterns. Together, they contribute to the increased prevalence of heart disease in groups with high pollution perception. This also suggests that improving environmental pollution such as noise and paying attention to individuals' psychological states are of great significance for the prevention of heart disease.

### 4.2 Perceived noise pollution and prevalence of other cardiovascular diseases no significant association

The results of this study showed that the prevalence of heart disease was higher in the group with a higher perception of noise pollution, but there was no significant association with the prevalence of hypertension, dyslipidemia, stroke, or overall cardiovascular diseases. The results of this study are inconsistent with previous research (46–48), all of which found that noise pollution is an important contributing factor to cardiovascular diseases such as hypertension (46), dyslipidemia (47), and stroke (48). The results of this study can be comprehensively explained by the following reasons.

Firstly, due to differences in the pathological mechanisms of various cardiovascular diseases, the association between heart disease and the perception of noise pollution may be stronger, while other cardiovascular diseases are dominated by multiple factors, and the impact of noise pollution may be masked by other factors. Noise pollution may directly affect cardiac function through psychological stress (such as anxiety and sympathetic nerve excitement) (49). Long-term stress can lead to a reduction in heart rate variability, an increase in myocardial oxygen consumption, and even induce arrhythmia or coronary artery spasm. These mechanisms are directly related to the onset of heart diseases (such as coronary heart disease and arrhythmia) (49). In contrast, hypertension and dyslipidemia are more related to physiological and metabolic factors (such as diet, obesity, and genetics); stroke is affected by multiple factors such as hypertension, arteriosclerosis, and thrombosis. Therefore, the impact of noise pollution may be masked by these more critical factors, or may only become apparent after long-term exposure. In addition, “perception”, as a subjective indicator, may not have accumulated to a level that triggers a significant association.

Secondly, the particularity of noise pollution perception may mask the association between noise pollution and the prevalence of cardiovascular diseases. This study focuses on the perception of noise pollution, emphasizing subjective experience rather than objective noise decibel values. It is possible that only when the perception of noise reaches certain intensity will it have a significant impact on the heart, while its impact on other diseases has not yet exceeded the “threshold”, so no statistical association has been observed. Finally, limitations in sample size and confounding factors, among other things, may have obscured this potential association. The sample size included in this study is relatively small, which may fail to detect the weak association between noise pollution and the prevalence of cardiovascular diseases. In addition, although this study has controlled for variables such as age, BMI, and SES, these variables have a stronger association with cardiovascular diseases such as hypertension, dyslipidemia, and stroke, which may weaken the independent impact of noise perception on these diseases.

### 4.3 Discussion on controlled variables

Regarding the relevant control variables, this study found that age, place of residence, educational level, BMI, and SES are associated with the prevalence of cardiovascular diseases among residents to varying degrees. Firstly, this study revealed that the prevalence of cardiovascular diseases in residents gradually increases with age, which is consistent with previous studies (50, 51). This pattern is mainly caused by multiple factors, including the decline in the body's physiological functions, reduced metabolic capacity, cumulative effects of chronic diseases, as well as lifestyle and environmental factors. Secondly, this study found that the prevalence of stroke was lower among rural residents than among urban residents, which was inconsistent with some previous studies (52, 53). The inconsistency may be attributed to differences in sample sources between this study and previous studies, including variations in specific urban–rural regions, economic levels, and age structures. In addition, stroke diagnosis may rely on clinical symptoms, imaging examinations (CT/MRI), or self-reporting. Previous studies adopted stricter imaging confirmation, while this study relied on self-reported diagnosis, which may lead to differences in the statistical results of prevalence rates.

Thirdly, this study found that groups with higher education levels have a lower prevalence of hypertension and a generally lower prevalence of cardiovascular diseases. The results of this study are similar to those of previous studies (54, 55), suggesting that special attention should be paid to groups with lower education levels in the prevention of cardiovascular diseases. The main reason is that groups with higher education levels usually have easier access to scientific health information, and thus are more likely to adopt healthy lifestyles. For example, they tend to follow a low-salt and low-fat diet and reduce the intake of processed foods; maintain regular exercise to control weight; take the initiative to quit smoking and limit alcohol consumption, thereby reducing exposure to risk factors. In addition, groups with higher education levels often have more stable occupations and higher income levels, enabling them to afford better medical services (such as regular medical visits and the purchase of antihypertensive or lipid-lowering drugs). Moreover, they have stronger compliance with medical advice (such as strictly taking medicines as prescribed by doctors), which reduces the deterioration of diseases caused by delayed or irregular treatment.

Fourthly, this study found that there is a significant positive association between BMI and the prevalence of cardiovascular diseases among residents, and the results of this study are similar to those of previous studies (56–58). All the aforementioned studies (56–58) indicate that BMI is an important risk factor for the occurrence and development of cardiovascular diseases. Finally, there is a significant negative association between SES and the prevalence of cardiovascular diseases, that is, the higher the SES, the lower the possibility of suffering from cardiovascular diseases. The results of this study are similar to those of previous studies (59, 60). Groups with high SES usually have higher education levels and stronger health literacy, enabling them to more easily understand scientific health knowledge and take the initiative to translate it into actions. In addition, high income and stable occupations usually mean more comprehensive medical insurance coverage or self-payment ability, allowing them to afford services such as regular physical examinations and specialized diagnosis and treatment, so that they can detect the precursors of cardiovascular diseases early and implement interventions.

### 4.4 The significance of this study for public health policy formulation

This study provides a multi-dimensional scientific basis for public health policy formulation, and has clear practical guiding value, especially in the prevention and control of noise pollution and health intervention for key populations. The specific significance is as follows:

Firstly, this study confirms that residents' high perception of noise pollution is significantly related to the increase in the prevalence of heart disease, which suggests that policy formulation needs to break through the traditional idea of “only relying on objective decibel data” and incorporate residents' subjective perception into the noise management system. We can specifically strengthen the noise monitoring and management in areas with high frequency of residents' activities (such as residential areas, school surroundings, and community parks). For example, strictly control the time and intensity of traffic noise and construction noise, and promote technical measures such as low-noise pavements and sound insulation barriers. In addition, combined with the differences in noise perception among residents in different regions, formulate differentiated prevention and control standards to avoid the limitations of the “one-size-fits-all” policy.

Secondly, this study identifies several key factors affecting cardiovascular diseases, providing a basis for policies to accurately target intervention objects. For groups with low education levels, it is necessary to popularize health knowledge such as low-salt diet and regular exercise through popular forms such as community health lectures and short video science popularization, and simplify the way of conveying medical advice to improve their treatment compliance. For groups with high BMI, we should promote the construction of community fitness facilities, carry out weight loss intervention programs, and restrict the excessive marketing of high-sugar and high-fat foods, so as to work together from the environmental and behavioral aspects. For groups with low SES, it is necessary to improve the coverage of medical insurance, reduce the out-of-pocket ratio of antihypertensive and lipid-lowering drugs, and provide free or low-cost physical examination services to reduce disease delays caused by economic reasons.

Finally, this study suggests improving the public health system linked by “environment-behavior-health”. The study reveals that noise pollution affects heart health through physiological stress, psychological anxiety and behavioral changes (such as reducing exercise), suggesting that policies need to build a multi-departmental coordination mechanism. It is suggested that the environmental protection department and the health department jointly establish a “noise pollution-health impact” monitoring network and regularly issue regional health risk warnings; it is suggested that the education department incorporate noise protection and cardiovascular health knowledge into primary and secondary school courses to cultivate the public's awareness of environmental health from the source; it is suggested that priority should be given to ensuring the “low-noise design” of residential areas in urban planning, such as reasonably arranging the distance between traffic arteries and residential areas, reserving green isolation belts, and at the same time supporting the construction of indoor sports venues to alleviate the problem of reduced exercise of residents in high-noise environments.

In conclusion, this study incorporates subjective perception into the framework of environmental health research, emphasizing that policies need to take into account both “objective environmental governance” and “individual subjective experience”. Through targeted noise prevention and control, precise intervention for key populations, and linkage of multi-department resources, the burden of cardiovascular diseases can be effectively reduced, and public health policies can be promoted to transform from “disease treatment” to “risk prevention”.

### 4.5 Limitations of this study

While this study has preliminarily clarified the association between Chinese adults' perception of noise pollution and the prevalence of cardiovascular diseases, offering certain reference directions for formulating public health policies, there remain some aspects that need improvement. First, the core independent variable of this study was subjective perception of noise pollution. We did not perform objective noise level (dB) measurements, nor did we assess participants' hearing status. Consequently, discrepancies may exist between subjective perception and actual environmental noise exposure, and hearing ability may influence perceived outcomes, leading to recall bias and self-report bias, which may compromise the accuracy of the results to some extent. Second, constrained by the variable set in the CGSS 2021 database, this study was unable to sufficiently adjust for key confounding factors, including smoking, alcohol consumption, diabetes, occupational exposure, air pollution, and urbanization level. These factors may affect the association between noise perception and cardiovascular health, potentially resulting in residual confounding and reducing the stability of effect estimates. Third, this study adopted a cross-sectional design, which can only reveal associations between variables but cannot establish temporal order or determine the causal direction between noise perception and heart disease. Fourth, the diagnosis of cardiovascular diseases (especially stroke) in this study relied on self-reporting by respondents rather than imaging confirmation such as CT or MRI, which may lead to misdiagnosis or underreporting of diseases. Meanwhile, although this study found a lower prevalence of stroke among rural residents, it did not incorporate key indicators such as objective urban–rural noise levels, healthcare accessibility, and diagnostic rates, making it difficult to fully explain the mechanism underlying the urban-rural difference. Finally, this study did not obtain provincial average noise level (dB) data across China, so stratified comparisons between objective noise and subjective perception could not be performed. In addition, the sample size and geographical coverage may limit the statistical power to detect weak associations.

Based on the above limitations, we propose the following prospects for future research. First, future studies should combine on-site noise monitoring (dB) with subjective perception scores and assess participants' hearing status. This will allow systematic comparison of the associations between objectively measured and subjectively perceived noise pollution and cardiovascular disease prevalence, thereby improving the reliability of the results. Second, future studies should include more comprehensive confounding variables, such as smoking, alcohol consumption, chronic diseases, air pollution, urbanization, and healthcare utilization. Third, longitudinal designs such as cohort studies are recommended to clarify temporal relationships, reduce reverse causality bias, and enhance the ability of causal inference. Fourth, it is recommended to use physician diagnosis or imaging examinations instead of simple self-reporting of cardiovascular diseases to improve the accuracy of disease definition. Finally, stratified analyses should be conducted for urban and rural areas. Combined with objective noise levels, lifestyle, medical conditions and other factors, future research can further explain urban–rural and provincial differences, and provide more precise evidence for formulating regional noise prevention and cardiovascular disease intervention strategies.

## 5 Conclusion

This study, based on the CGSS 2021 cardiovascular disease dataset, explored the association between Chinese adults' subjective perception of noise pollution and the prevalence of cardiovascular diseases. The results showed that the prevalence of heart disease in the group perceiving high noise pollution was significantly higher than that in the group perceiving low noise pollution, indicating a significant positive association between noise pollution perception and the prevalence of heart disease. However, the study found no statistically significant association between noise pollution perception and the prevalence of overall cardiovascular diseases, hypertension, dyslipidemia, or stroke. Based on the above results, this study emphasizes that when formulating strategies for noise pollution prevention and control as well as cardiovascular disease prevention, attention should be paid not only to objective noise data but also to residents' subjective perceptions. Nevertheless, due to the cross-sectional nature of this study and limitations in sample size and data collection methods, further longitudinal studies are needed to verify the causal relationship and long-term impact between noise pollution perception and cardiovascular diseases.

## Data Availability

The raw data supporting the conclusions of this article will be made available by the authors, without undue reservation.
